# Variations in the Development and Density of Tooth Tissue

**Published:** 1903-06-15

**Authors:** J. Taft


DENTAL REGISTER.

Vol. LVII. June 15, 1903. No. 6.

### VARIATIONS IN THE DEVELOPMENT AND DENSITY OF TOOTH TISSUE.

BY J. TAFT, M.D., D.D.S.

Read before the Southwestern Michigan Dental Society, April 8, 1903.

This caption implies that there is a diversity in the density of the teeth of different persons, and in the same person at different periods of life, and indeed apparently under nearly uniform environment. But much more is this apparent in the ever-varying vicissitudes to which humanity is subjected.

Doubtless, heredity, which is the transmission of physical and mental conditions, defects and peculiarities, from one generation to another, has much to do in the production of defective structure of the teeth of mankind. Variations in this transmission often occur because of dissimilarities between progenitors.

How often is it that parents are unlike in physical make-up, and this is shown in their teeth as pronouncedly as in any other tissue. If the teeth of both parents are alike defective, those of the offspring may be equally so, and are often worse, or one parent may have good and the other defective teeth, and the offspring may then partake of the quality of one or of the other. The results that sometimes occur under such circumstances are very peculiar and striking indeed. Further on this line I would suggest that the subject of heredity is worthy of more attention, study and investigation than has generally been given to it, especially in regard to the teeth.

That the teeth vary so much in their density is shown in a variety of ways. The close observer in his daily work will have ample evidence of variations in density that obtain in the teeth of different persons, and in the same person at different periods of life. In the normal, uninterrupted growth and development of the teeth up to twenty-five or thirty years of age they increase in hardness, and in some instances even five to ten years beyond this. In all cases the permanent teeth are easily cut with the excavator or drill for five or ten years after eruption; this is true even of the better varieties of teeth. The readiness with which some fracture under percussion or blows from a hammer, or from gradual pressure, as in a vise, when compared with those of firmer texture, show a radical difference in solidity and firmness.

The examination of the sections of teeth with the microscope shows a condition of the tooth structure quite divergent from the normal. The varieties that appear in the hard tissues of the teeth are shown in the enamel rods, in the defective calcification, rendering them less dense than normal. In the well-formed enamel the rods converge to a central point in the crown of the tooth. But in the defective enamel this convergence does not obtain, but a divergence to a greater or less extent is present. And furthermore, the union of the rods is much less firm in the defective teeth. There is quite a variety in the surface of the enamel, in some it presents a smooth and highly-polished surface, while in others there is a more or less pronounced want of the bright and glistening appearance seen in the more perfect teeth; indeed they sometimes have a rough and corroded surface. The canals in the normal dentine, like the enamel rods, converge to a common center in the crown of the tooth, but a perfect arrangement of them is quite exceptional.

They are frequently many degrees out of proper inclination and being much more curved than normal. In some cases the canals are greater in number in a given area than normal, while in other instances they are less in number, so precluding the formation of perfect tissues. This abnormality consists in part at least of irregularity in the arrangement of of the canals through the dentine, and defective adjustment of the canal rods.

There are often found in the dentine defective areas where there is imperfect calcification and a deficiency in the proper supply of canals; these areas are called interglobular spaces, and when attacked by decay go very rapidly. The dentine and enamel are made up of organic and inorganic material; the former first takes on organic form and arrangement, in other words the odontoblasts and net-work that constitute the matrix in which the lime salts are deposited are arranged in accord with the typical form for normal dentine. It should be kept in mind that in these developing processes interruptions may and often do occur; these interruptions vary greatly in degree and extent. These interruptions or defective areas are sometimes very limited in extent, affecting only a mere spot, and in others much greater in out-reach, involving in some cases the entire body of the dentine and of the enamel as well. The following are some of the conditions under the influences of which these defects occur:

*Faulty Nutrition:* This may occur from an insufficiency of food, or from inappropriate food, or its improper preparation. In illustration of this statement it may be said that a large proportion of our bread supply is in its preparation almost wholly deprived of its bone-making material. The lime salts are by the bolting process removed with the brans, the starch and gluten being retained, these constituting the flour of which ninety percent of our bread is made, with which is it impossible to make the most nutritious bread. The question of the presence of lime salts in food is a very important one, one with which those who prepare our food should be thoroughly familiar, and especially is this true of those who prepare food for the supply of infantile life. Another very important question is the character of the food supply, which the mother receives during the period of lactation. Those who have given close attention to the subject inform us that very few mothers afford the children in infancy food supplied with bone phosphate in sufficient quantity to meet the demands of the growing bone structures of the body, and especially of the teeth. This occurs, of course, because of an insufficient supply of proper food by the mother.

In this respect there is a very common deficiency, indeed, in many instances the bony tissues of the child are literally starved by this privation. The child is not the only sufferer in this direction. The welfare of the mother is jeopardized as well. Nor are these things the only points of danger. The digestive function of either the mother or child, or both, may be defective, and if of the mother only, it is impossible that she could afford proper nutrition for her child. The digestion of the mother may be good, but that of the child faulty, when the processes of digestion and assimilation will be defective, and then faulty structures will necessarily be made. Good hygienic conditions must be afforded to both mother and child, if the growing and developing processes are to bring about strong and normal tissues.

All the conditions above named may be perfect, and yet disease of some form or other may step in and interrupt or destroy the processes of development and growth more or less persistently. We will not attempt in this paper to discuss these further than to say that any and all diseases of the general system which are inimical to the proper organization and growth of the structures of the body especially, will apply to the teeth. Affections of the skin and mucous membrane of the mouth and alimentary canal, such as measles, scarlatina, small-pox, diphtheria, stomatitis and other dermal affections, are most injurious to growing teeth, and are quite likely to injure the teeth during the time of their calcification.

These few suggestions may give us some intimation as to what should be done if we would secure the best bony tissues applying these principles to the teeth as well as to other hard structures. Oftentimes local affections spring up in the mouth and its adjacent parts, that are injurious to the growing teeth. The development of the organic structures of the teeth is a very intricate and delicate process. The formation of the odontoblasts may be easily impaired, and these organs being defective, a perfectly-developed structure is impossible. Even when these are well formed and properly developed, failure to secure good calcification may occur from a faulty condition of the lime salts while in the plasm, or from a want of energy in the calcifying process, viz: the precipitation and arrangement of the inorganic material in the matrix already prepared for it. For perfection in this work a certain balance is indispensable to secure the best results.

Considering all these things, may we not begin to understand how easy it is for faulty structures to be made, and how many embarrassments there are that stand in the way of structural perfection?

In addition to the inferences that may be drawn from the above considerations, I will venture for the remainder of this paper to draw upon the experience and observations of a few noted practitioners, who have had large experience in treatment for securing good bone tissue, and especially that for the teeth. These quotations have reference mainly to supplying the material requisite for building bone tissue, by the artificial administration of lime salts in the form of bone phosphate. The use of bone phosphates for securing good dental tissue apparently receives less attention now than in former years. It is, however, largely used by physicians in the form of phosphate syrups for the various general affections, especially those of the nervous system, and of the lungs and other vital and functional organs.

It is stated in Championniere's Journal of Pract. Med. and Surg., that "Dr. Lavau, of Birac, called the attention of the Academy of Medicine of Paris to the importance of sulphuret of lime in the regeneration of bony substance. Mr. Lavau observed, more than twenty years ago, that this agent diluted in olive oil and used in frictions' to destroy itch induces enlargement of the joints of the fingers. This observation led him to prescribe frictions with sulphuret of lime on the head of rickety subjects whose fontanelles were excessively large, or persisted beyond the normal period, and he obtained with surprising rapidity the ossification and obliteration of these membranous apertures. Mr. Lavau therefore surmises that the same treatment may perhaps be also applicable to the secretion of the periosteum in the great process of the reproduction of bone." If such is the case, this agent might probably prove useful in promoting the deposit of dentine over exposed and sensitive pulps.

In the course of a very interesting paper on the medical properties of the alkaline hypophosphites, Mr. Jno. Taylor, speaks thus of their relation with dent tion: "The impulsive demand for tribasic phosphate of lime in the construction of the teeth contributes to the disturbing influence called the fever of dentition; and this disturbance is often found to be most pyrexial in children that have been ill fed, or that have been too long suckled, both instances showing the want of a due proportion of phosphates. In the robust there is sympathetic spinal irritation, tending to convulsion; in the feeble and cachectic, sympathetic nausea and purging, wearing-out existance. In both forms have I given hypophosphites of potash with marked success; in the first, or asthenic form, with solution of acetate of ammonia and syrup of rhubarb; in the latter with acacia and some tonic or aromatic tincture. It is delightful to witness the consolatory effect upon a fractious, pseudo-inflammatory child, some cases of minor intensity being cured by the first effort, say eight grains of the hyphophosphites in a two-ounce mixture. Those of the asthenic form had considerable dyspnoea, cough, pale dry skin, and a feeble pulse; some requiring ammonia as an adjunct; others not; but in all the impressive power of the hypophosphites, with its appropriate adjunct, was most satisfactory—frequently simple syrup or mucilage sufficed.

*The absence of phosphate of lime in mother's milk a cause of infant mortality.* "The Courrier, of Paris, in a very able article on the mortality of infants, attributes it in a great many instances to the insufficiency of the development of bone, and adds that the milk of a healthy nurse ought to contain two and one-half grammes of phosphate of lime, which is the basis of all osseous matter. From observations made, it appears that scarcely one in ten women has milk coming up to this standard, and, therefore, the infants, it is said, necessarily perish or grow up sickly, and probably deformed."—*Med. Record* and *Boston Med. and Surg. Journal.*

*Phosphates of lime, its assimilation and therapeutical employment.* "We notice in Le Mouvement Medical (No. 17), a summary of a recent published Memoire of MM. Dusart et Blanche, who have made a number of experiments upon animals and men, to ascertain the action of the gastric juice upon phosphate of lime. They find that much of the phosphate of lime of commerce is nothing more than carbonate of lime, and to this cause they attribute the varying experiences of different observers.

"They have found that the hydrated phosphate of lime, and recently precipitated, is the most suitable for assimilation; and in their experiments they employed the lacto-phosphate of lime, the ultimate result of the action of gastric juice upon phosphate of lime. They made some experiments on Guinea-pigs, in whom they produced fractures, and they found that the increase of weight of the bones of such of the animals as were submitted to the action of the phosphate of lime exceeded that of others placed under ordinary regimen more than thirty-three percent, though all the animals were given exactly the same quantity of aliment.

"They administer two grains of the lacto-phosphate of lime to the ounce of syrup, daily, in soup.

"Dr. Perate has found this lacto-phosphate of lime extremely beneficial in dyspepsia, from insufficient secretion of the acid of the gastric juice."

"For a long time past the calcareous salts have been employed in medicine. The numerous experiments of Chossat, who established in a vigorous manner the fact that certain animals did not find in their ordinary food sufficient mineral substance for the reparation of losses of osseous structure, have increased considerably the uses of these agents. It is evident, in fact, that in order for the physiological equilibrium to be maintained, alimentation must be completed by addition of calcareous material to keep up the solidity of the skeleton, and to prevent the bones from becoming fragile.

"In cases of rickets and osteomalacia, Bouchut prescribed phosphate of lime in quantities of from two to five grammes in the day. Piorry gave this agent to children in doses varying from five to ten grammes, it having been reduced to an impalpable powder and mixed with a soft substance as cream or marmalade. In many cases this physician served the phosphate of lime with iodide of potassium. Guersant and many others prescribe eight to ten small pastilles to be taken every day, each containing three centigrammes of lactate of iron and five centigrammes of phosphate of lime. In their observations on men and the lower animals, MM. Gosselin and A. Milne-Edwards made out that in cases of fracture the necessary time for consolidation was notably shortened after the administration of phosphate of lime with the food.

"A book has lately been published in Paris, by M. Dusart, on the physiological and therapeutical properties of phosphate of lime. The author maintains, after numerous experiments in the animal kingdom, that this salt is the natural exciting agent in the functions of nutrition; that it induces the albuminoid matter to assume the cellular shape, and that it controls the formation of tissues. In short, according to M. Dusart, phosphate of lime is eminently an agent of nutrition. This view holds good, also, in respect of the vegetable kingdom., and the author asserts that the salt in question is concentrated in the leaf-bud, but is almost absent from the fully-developed leaf, so as to become concentrated in the seed preparing for the ultimate development of the embryo. M. Dusart points out that the phosphate of lime is always conjoined with nitrogenous matter in plants, and that the relative proportion of the salt and the nitrogen is always identical wherever they are found. In animals the same phenomena take place, and, when they are made to feed much upon the phosphate, they absorb more food, and increase rapidly in weight, owing to the transformation of the albuminoid matter contained in the food into muscular fiber."—*Lancet.*

Cases Cited.—Mr. J., aged twenty-two, sanguino-bilious temperament. Has twenty-eight permanent teeth erupted. They are full sized, well formed in shape, good color, and thoroughly calcified for his age, with perfect blending of enamel caps, and no sign of decay about them. An older brother has a perfect denture in every respect. There are other brothers and sisters who are said to have as good teeth as these which I have examined.

The father and mother of this family have hardly any teeth left in their mouths. They have been decayed many years; consequently these children would inherit poor teeth. Now for the hygiene which prevented that calamity. During the infancy of some of the children, and before the two brothers, of whose teeth I have particularly spoken, were born, the parents moved to a new State, in the West, and were very thankful to get their wheat ground into meal for bread, as in the first settlement no bolts were put in the mills. A taste for unbolted bread was acquired, which has continued to the present day, and no fine flour has ever been used in the family excepting on extra occasions. The family have always enjoyed remarkably good health, which they attribute to their bread diet. Noticing a slight furrow across the labial surface of Mr. J.'s, upper central incisor, I remarked to him that he must have had a fit of sickness when between two and three years old. He said he did not know, but would ask his mother. His mother confirmed my conjecture, saying that he was sick about six weeks at the age above named.

Miss H., aged twenty, has erupted twenty-eight teeth, nearly all decayed. The teeth have a pearly tint and are badly calcified; the sound dentine cuts easily. I remarked to her that her "teeth looked as though she lived on fine flour bread and butter." She laughingly said "that is the only kind of food I desire; I do live on it." She informed me that all her sisters had good teeth compared with hers; that they ate meat, potatoes, milk, etc.; that they laughed at her for her "old maid way of living."

Miss G., lymphatic temperament, aged thirteen. Has twenty-eight permanent teeth, pearly tint. The fissures in the crowns of the molars and bicuspids are decayed, as are also many of the proximal surfaces. The teeth cut soft and indicate mal-dentification. I plugged fourteen cavities in the teeth of this girl whose father is a miller. The family uses the finest of flour. This girl was nursed on milk made from fine flour, and her teeth were starved for the want of of phosphate of lime."

The greatest mischief ensues to the teeth when the phosphate of lime is denied to the embryo infant. It does not replenish the blood of the mother with this substance, as it naturally occurs in the food, the forming teeth of the child must inevitably suffer and the mother's also. When we reflect on the large quantity of lime salts necessary to build up the bony tissues of a child until it is eighteen months old, and the waste of the same material which is daily excreted from the body of the mother and child, we may cease to wonder at the universal decay of teeth in Americans, and smother the sacrilegious inquiry "Why did not the Creator make the teeth to last as long as the rest of the body?" There is no need of relating more cases. I could do so by the score, for I have inquired of many people in regard to their habits of life in relation to the teeth, and the story is nearly always the same.

DISCUSSION.

Dr. J. H. Palin, of Grand Rapids: I have no doubt much can be done in the way of producing by proper hygienic practices a better grade of tooth tissue. I have a theory that I am putting into practice with my own children. I instruct my boy who is five years old to thoroughly pulverize with his teeth, every day, three grains of corn, using both sides of the mouth. I hope by the exercise of the deciduous teeth to develop the jaws and permanent teeth, because of the increased nutritive pabulum brought to the regions of developing teeth.

Dr. Geo. Cook, of Chicago: Dentistry in its practice is looking nowadays to prophylactic rather than therapeutic treatment. The only practicable way of accomplishing worthy aims is to see to it that the better quality of tooth tissue is developed. Osmotic pressure will govern the amount of pabulum the cells will appropriate as nourishment. Massage or exercise are good methods of accomplishing this. The cells then will take up all the organic material they can retain and build it into good, hard tooth tissues from its soluble condition. The enamel and odontoblast cells are both made more active because of local exercise. Muscular and bone strictures develop because of exercise; why should not the teeth? Whilst tooth building is a chemico-physiological phenomenon, its function may be wonderfully increased by proper exercise. Dr. Black's investigation seems to indicate that the teeth are never hard, nor soft. It may be that Dr. Block's findings have not been properly interpreted; but I am convinced that some teeth are harder than others, and also that the same teeth differ at different periods. The paper is timely and ought to be carefully considered. I trust we may some time know how to produce the best tooth structure by hygienic or dietary methods.

Dr. R. W. Morse, of Lansing: It has occurred to me that the extensive use of prepared foods at the present time is destined to have some place in at least affecting or modifying the development of the tooth tissues. I am not certain that we can change or modify the already-formed tooth structure, particularly the enamel, whose generative organ has been lost. We may fortify it, or support it with a greater development of dentine which is deposited continually from a more highly-organized and persistent organ of development. The dental tissues seem to harden with age. Can we not therefore imitate nature's method and produce this hardening at an early age by some form of artificial exercise or system of feeding? Enamel defects that are congenital may be avoided by advising every mother as the necessity of taking proper exercise during the fœtal life of the child.

Dr. J. M. Thompson, of Detroit. We can't give the subject of feeding mothers to secure good tooth structure too much attention, as it is fundamental to our solution of the dental problems. A case has come under my observation of where no attention was given the mother in this respect, and the child which was born so illy developed that it lived but eleven months. A second child born to the same mother is a finely-developed specimen of what may be done when care is taken to secure good bone and muscle development.

Dr. Taft: I fully sympathize with what has been said in regard to masticating the food thoroughly, and also with the remarks on exercise, etc. Faulty mastication and incomplete insalivation of the food is the great bane of modern civilization. If the food elements are not in the pabulum no amount of massaging will be of value in securing good tooth tissues. The tissues must have the elements needed for building the structure in a constant as well as a perfect form. If the required lime is not at all times present in the nutritive pabulum the tooth tissues will not be structurally uniform; pits and other defects will show themselves. No brick and all mortar will build a poor wall or house. If the tissues are not built homogeneously and completely at the time of calcification, the opportunity for changing the condition is forever lost.

It is said that 45 percent of human beings die before they are five years old. If any such proportion of deaths occurred among the cattle of the stockman he would immediately start an inquiry as to the cause and adopt proper methods for overcoming this enormous fatality. We do not seem yet to be awake on this subject. If the food material is lacking at the proper time we can't expect a perfect tooth, or if there is a lack of function the tooth may be abnormal.

